# Comparative genomic analysis of Chinese *Erwinia amylovora* strains reveals genetic variations, plasmid diversity, and potential common ancestor

**DOI:** 10.1002/imo2.44

**Published:** 2024-11-29

**Authors:** Peijie Gong, Weibo Sun, Jianping Yi, Jian Han, Meili Liu, Chenyang Han, Liang Ming, Bangwei Wang, Ming Luo, Yancun Zhao, Fengquan Liu

**Affiliations:** ^1^ Institute of Plant Protection, Jiangsu Academy of Agricultural Sciences, Jiangsu Key Laboratory for Food Quality and Safety State Key Laboratory Cultivation Base of Ministry of Science and Technology Nanjing China; ^2^ Technical Center for Animal, Plant and Food Inspection and Quarantine of Shanghai Customs Shanghai China; ^3^ College of Agronomy Xinjiang Agriculture University Wulumuqi China; ^4^ Center of Pear Engineering Technology Research, State Key Laboratory of Crop Genetics and Germplasm Enhancement, College of Horticulture Nanjing Agricultural University Nanjing China; ^5^ College of Plant Protection Nanjing Agricultural University Nanjing China; ^6^ Department of Plant Pathology/Key Laboratory of Agricultural Microbiology, College of Agriculture Guizhou University Guiyang China

## Abstract

The bacterial pathogen *Erwinia amylovora* is the causative agent of fire blight, a devastating disease affecting rosaceous plants globally. Pathogenicity tests in immature pears demonstrated *E. amylovora* discovered in China exhibit different virulences. Through full‐genome sequencing and genetic variations assay, Each strain exhibited a chromosome size of approximately 3.8 Mb, with distinct distribution patterns in plasmids. The genomes of all sequenced strains were compared to the model strain CFBP1430. Single‐nucleotide polymorphisms (SNPs) and short deletions, insertions, and other polymorphisms (DIPs) were identified and shared SNPs and DIPs among strains suggest genomic homogeneity, with a higher level of specificity in DIPs. Phylogenetic analysis inferred that the primary pathway of fire blight spread to China followed a trajectory from the Middle East to Central Asia, ultimately reaching Xinjiang.

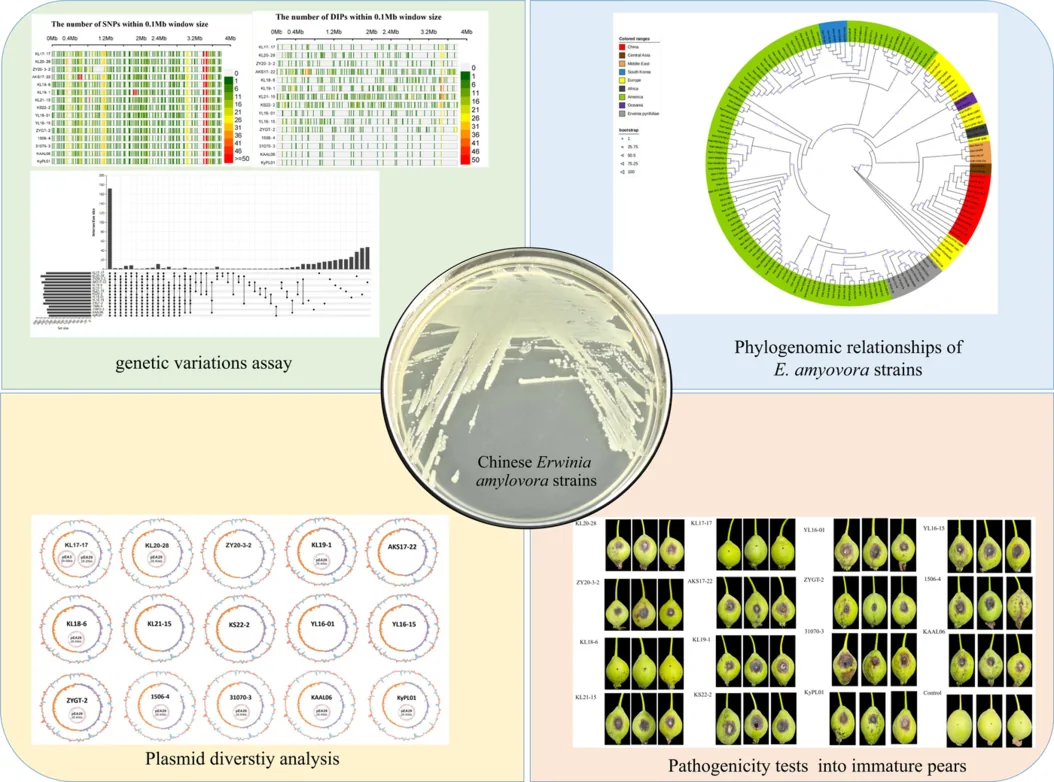

To the editor,

Fire blight, a highly destructive disease that impacts rosaceous plants on a global scale, is caused by the bacterial pathogen *Erwinia amylovora* [1, 2, 3, 4, 5]. Since the late 19th century, it was discovered that fire blight disease on pear is caused by *E. amylovora*, which is also the first bacteria in the history of science to be proven to cause plant diseases [6]. Over the past century, extensive research has been conducted on the pathogen *E. amylovora* from various aspects. Early studies mainly focused on its biological characteristics, while in the past 20 years, the research has gradually shifted towards the molecular mechanisms of pathogenesis [7, 8, 9]. The pathogen secretes virulence factors (also known as toxins) into the host, including the type III secretion system (T3SS) and exopolysaccharides [7, 10]. The T3SS is mainly composed of hypersensitive response, pathogenicity (Hrp), and hrp‐conserved (Hrc) proteins. These encoding genes are clustered on a pathogenicity island that contains approximately 60 genes related to virulence, including hrp/hrc domains, the Hrp‐associated enzyme region (HAE domain), the Hrp effectors and elicitors region (HEE domain), and the island transfer region (IT domain). Exopolysaccharides are a type of long‐chain polysaccharide that is secreted to the bacterial surface during growth [11]. Exopolysaccharides protect *E. amylovora* from dehydration and nutrient deprivation, facilitate bacterial infiltration into plant tissues, block plant vascular systems, and protect the bacteria from recognition by host defense responses [12]. The known virulence‐related exopolysaccharides include amylovoran and levan [13]. The proteins secreted by *E. amylovora* through T3SS include various effector proteins, including dspA/E, eop1, eop2, eop3, and eop4 (AvrRpt2EA), chaperone proteins dspB/F, and Hrp proteins (hrpN, hrpW, etc.) [9]. Among them, dspA/E and hrpN are important virulence proteins of *E. amylovora*.

The initial occurrence of fire blight in China was identified at Ili (Xinjiang) in 2015 and damaged apple production in 2016 severely [14]. It spread to the Korla region in 2017 causing significant damage to “Korla fragrant pear,” and subsequently spread to Gansu in 2020 [14]. To deepen our understanding of *E. amylovora* pathogenesis within China, we first conducted full‐genome sequencing of 13 strains isolated from various regions in China, as well as strains from Kazakhstan and Kyrgyzstan (Figure 1A; Table S1). Each strain exhibited a chromosome size of approximately 3.8 Mb, with distinct distribution patterns in plasmids. The genomes of all sequenced strains were compared to the model strain CFBP1430. Single‐nucleotide polymorphisms (SNPs), short deletions, insertions, and other polymorphisms (DIPs) were analyzed, showing shared SNPs and DIPs among strains with a higher level of specificity in DIPs. Additionally, a phylogenetic analysis was carried out to elucidate the evolutionary relationships among 132 *E. amylovora* strains and 10 *E. pyrifloiae* strains from diverse hosts and geographical regions worldwide. These findings revealed that all Chinese isolates shared a common ancestor and were closely related to a lineage comprising the Kazakhstan strain KAAL06 and the Kyrgyzstan strain KyPL01. Thus, we propose that the primary pathway of fire blight spread to China may follow a trajectory from the Middle East to Central Asia, ultimately reaching Xinjiang.

**FIGURE 1:**
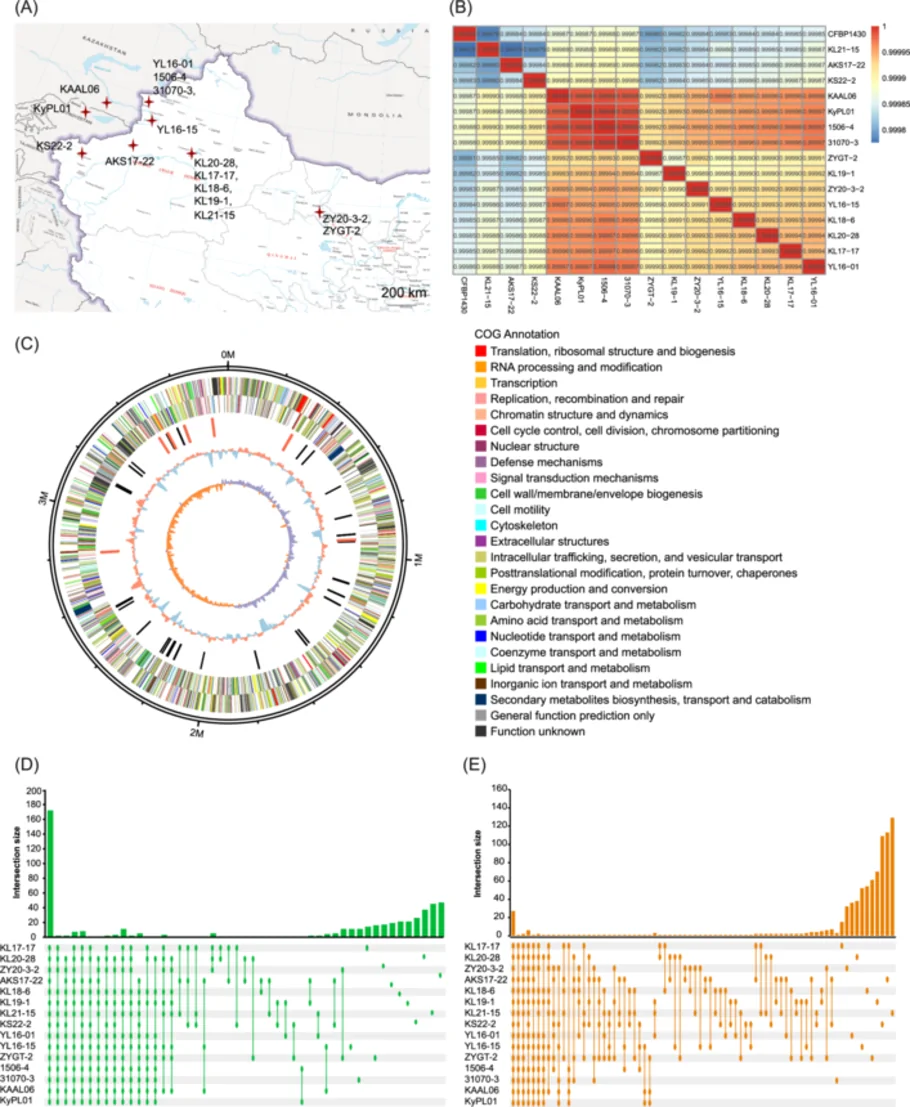
Whole genome sequencing and genomic diversity analysis of 15 *E. amylovora* strains. (A) The labeled regions represent the cities where the isolated strains were found. The map was created using the online tool at http://bzdt.ch.mnr.gov.cn/. (B) ANI values of sequenced *E. amylovora* strains. ANI was calculated using the Jspecies with MUMer algorithm. (C) The outermost circle of the genomic circular map shows the KL20‐28 genomic sequence position coordinates. Moving from the outer circle to the inner circles, they represent genes on the positive strand, genes on the negative strand, ncRNA (black for tRNA, red for rRNA), GC content (red indicates above the mean, blue indicates below the mean), and GC skew (used to measure the relative content of G and C in a circular chromosome, marking the start and endpoints; GC skew = (G − C)/(G + C); purple indicates above 0, orange indicates below 0. (D) UpSetR plots depicting the number of unique and shared differentially SNPs between each *E. amylovora* strain. Graph depicts the unique, total, and shared number of SNPs for each strain. (E) UpSetR plots depicting the number of unique and shared differentially DIPs between each *E. amylovora* strain. Graph depicts the unique, total, and shared number of DIPs for each strain.

## 1 RESULTS AND DISCUSSION

### 1.1 Genome assembly and features assessment of 15 *E. amylovora* strains

The complete genome sizes of the targeted strains (Figure 1A; Figures S1−S2) varied from 3,798,299 to 3,805,877 bp, with a genome GC content of 53.6% (Tables S1−S2). Protein‐coding sequences within the complete genomes of each strain were predicted by Glimmer 3.0 and ranged from 3376 to 3457 (Figure S2; Table S2). The ANI value of all 15 examined strains exhibited similarity levels exceeding 99.9% with the model strain CFBP1430 of *E. amylovora* (Table S3). Hence, all tested strains were identified as belonging to the *E. amylovora* species (Figure 1B). A comprehensive depiction of the genome characteristic for KL20‐28 was shown as an example (Figure 1C; Table S5). Additionally, genome‐seq comparison findings revealed a 99.87% similarity of the matched sequences in KL20‐28 and CFBP1430 (Figure S3).

### 1.2 Genes annotation and plasmids identification in 15 *E. amylovora* isolates

The predicted genes for each strain were annotated utilizing five databases, including COG, KEGG, GO, Swiss‐Prot, and NR. For instance, a total of 3403 genes of KL20‐28 were obtained functional annotations, representing 99.91% of the total genes (Table S4). Based on the COG (Clusters of Orthologous Groups of proteins) database, proteins were categorized into 25 classes (Figure 1C; Figure S4; Table S5). Based on the GO system (Figure S5; Table S6), cell categories showed a predominant role in cellular component with 2133 genes. Furthermore, 23 KEGG categories were clustered, in which carbohydrate metabolism (203 genes) was the top cluster (Figure S5; Table S7). We conducted a preliminary analysis of key factors related to pathogenicity, such as effectors dspA/E, eop1, eop3, AvrRpt2, and HopPtoC [9]; Harpin protein hrpN [7]; Chaperone protein dspB/F [9]; amylovoran synthesis cluster Ams [13] (Figure S11). The results showed that all the above factors were highly conserved in sequencing 15 strains and CFBP1430. Thus, except for a small difference in eop1 at the 3' end, the similarity of the remaining sequences is above 99.9%.

The conserved ~28 kb plasmid *pEA29* was shared among KL20‐28, KL17‐17, KL18‐6, KL19‐1, ZYGT‐2, 1506‐4, 31070‐3, KyPL01, and KAAL06, while no plasmid was detected in ZY20‐3‐2, AKS17‐22, KL21‐15, KS22‐2, YL16‐01, and YL16‐15 (Figure S6; Tables S8–S9). Moreover, a novel plasmid, named *pEA3‐like* (Tables S8, S10), was discovered in KL17‐17, which has not been documented in *E. amylovora* previously. The virulence factor genes in the *pEA29* plasmid were identified and annotated in 9 strains using the VFDB (Virulence Factors of Pathogenic Bacteria) virulence factor database [15]. The *pEA29* plasmid was found to be conserved in these 9 strains and was annotated to 4 identical virulence‐related genes, namely cheD methyl‐accepting chemoaxis protein CheD, tar/cheM ‐ methyl‐accepting chemotaxis protein II, pilW ‐ putative transposase, and pilW ‐ type IV fimbrial biogenesis protein PilW (Table S15). In addition, no virulence‐related genes were found in the plasmid pEA3 by VFDB analysis.

### 1.3 Comparative analysis of 15 sequenced strains with CFBP1430

A large‐scale chromosomal inversion was observed in all sequenced strains when compared to CFBP1430 (Figure S7; Table S11), ranging from 0.4 M to 3.7 M. Besides, a total of 5087 SNPs and 1996 DIPs were shared among these 15 strains (Table S12). 172 common SNPs (2580 in total) were identified among all strains (Figure 1D), constituting approximately 50.7% of the total SNPs. In terms of DIPs, 28 shared sites (420 in total) were identified (Figure 1E), representing approximately 21% of the total DIPs. DIPs exhibited a higher level of specificity within the genomes of each strain.

**FIGURE 2:**
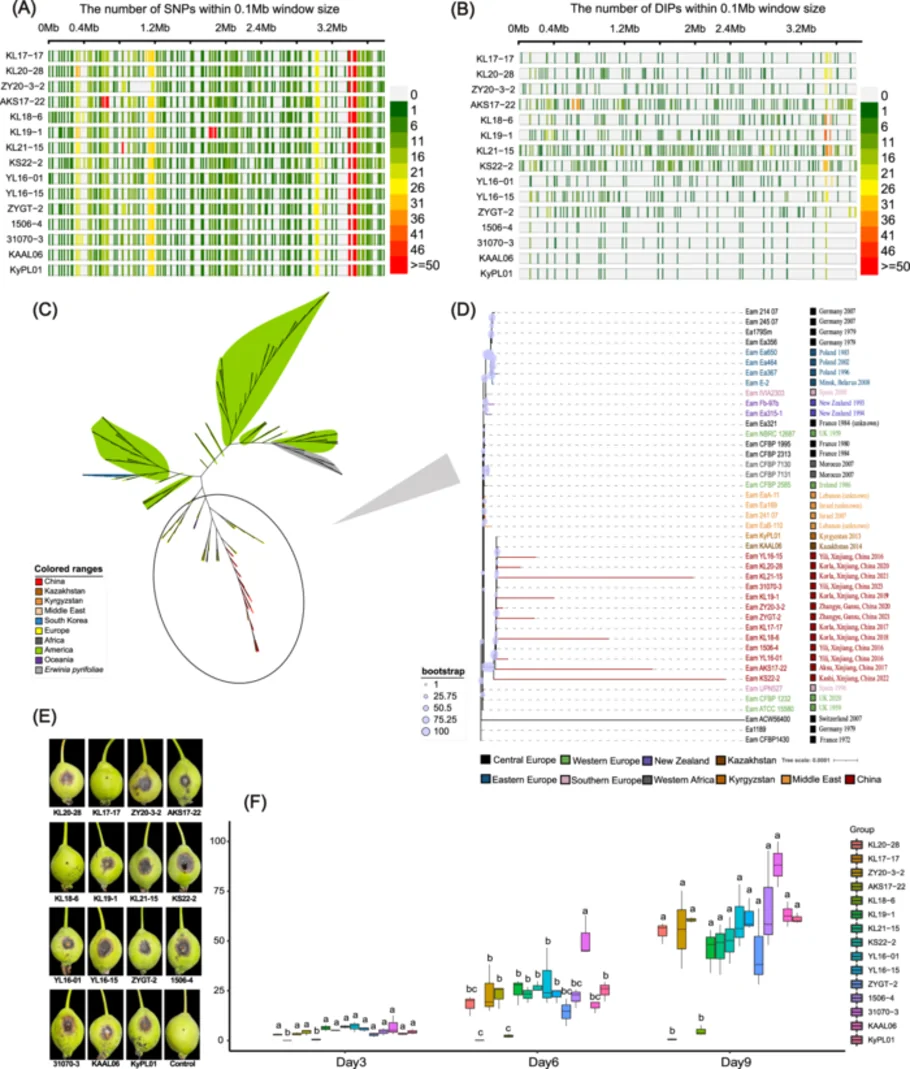
Chromosome density of single‐nucleotide polymorphisms/short deletions, insertions, and other polymorphisms (SNP/DIP) and traceability analysis of *E. amylovora* strains through whole genome sequencing and pathogenicity test. (A) The scale represents the size of the whole genome in megabases (Mb). The color code represents the frequency of SNPs and (B) DIPs occurring within 0.1 Mb. The fewer occurrences lean towards green (0 is represented in gray), while the more occurrences lean towards red (with a maximum value of 50). (C) Population phylogenetic analysis of *E. amylovora*. Unrooted tree revealed the relationship of 13 Chinese *E. amylovora* strains with other 132 *E. amylovora* strains and 10 *E. pyrifoliae* from various parts of the globe, using OrthoFinder2 through 2120 single copy orthologous genes of each stain. (D) Rectangular tree was generated by a core alignment of 43 *E. amylovora* strains from the unrooted tree, constructed using the maximum likelihood method (ML) with bootstrap test (based on 1000 replicates) through ITOL software. (E) Immature pear fruits were inoculated after 6 days with 15 sequenced *E. amylovora* strains. The negative control was treated with ddH2O sterilized water. (F) Virulence of the above‐mentioned strains was quantified in immature pear fruits at 3, 6, and 9 days postinoculation. The ratio of infection area for disease severity was calculated and assays were performed. All results are from representative experiments using ImageJ via color threshold quantification.

Besides, SNPs were distributed relatively evenly across the different strains, displaying a consistent density pattern (Figure 2A; Table S12). The distribution of DIPs did not exhibit a clear pattern, with the quantity of DIPs varying significantly across the strains (Figure 2B; Table S12). With the CFBP1430 genome as a reference to compare the genomes of *E. amylovora*, SNP variations were further investigated (Table S16) to provide information on mutations in genes that are possibly associated with the virulence of *E. amylovora*. These 15 sequenced strains showed SNP variants in pathogenicity‐related genes belonging to bacterial secretion system, flagellar assembly, bacterial chemotaxis, plant‐pathogen interaction, and biosynthesis of secondary metabolites [16]. Notably, 32 SNPs were found to be specific to these 15 sequenced strains, including 2 synonymous and 30 nonsynonymous mutations (Table S17).

These 15 sequenced strains showed a genome‐wide sequence similarity of over 99.98% compared to the reference strain CFBP1430. However, the majority of the chromosomes experienced inversion events, which is consistent with previous reports [16]. Although the whole‐genome structures of the *E. amylovora* strains within China are almost identical at present (with the presence of SNPs or small fragment variations), it is not ruled out that in the distant future, the occurrence of more strains resulting from chromosomal recombination may lead to the emergence of differences in pathogenicity among strains.

### 1.4 Chinese strains have a common ancestor and may originate from Central Asia

Combining the genomic reference information of 132 *E. amylovora* and 10 *E. pyrifoliae* strains in NCBI with the 15 sequenced strains in this study, a phylogenetic tree was constructed and showed that all strains were broadly divided into two categories (Tables S13−S14). One category mainly belonged to the strains from the North American, while the other branch consisted of a relatively complex, including the 13 Chinese strains (Figure 2C; Tables S13−S14). Five *E. amylovora* strains isolated in Korea were closely related to the American strain UT5P4, consistent with previous study that the Korean strains were introduced from the Americas [16]. Our analysis showed that 13 sequenced Chinese strains were clustered in the same subbranch, suggesting a common origin for these strains. Furthermore, the Chinese strains showed the closest clustering with strains from Kazakhstan and Kyrgyzstan, which were also more closely related to strains from the Middle East, followed by strains from European regions (Figure 2D). A further phylogenetic circular tree revealed the relationship of 15 *E. amylovora* strains in this study, among 255 other *E. amylovora* strains from various parts of the globe. The results are consistent with the description above (Figure S8). However, all strains in South Korea were clustered together with the American strain UT5P4, further away from the Chinese strains and other strains in Asia. Therefore, we infer that the transmission of pear disease to China may come as following route: Europe → Middle East → Central Asia → Xinjiang.

Since the early 21st century, several neighboring countries of China, including Kazakhstan (2008), Kyrgyzstan (2008), and Korea (2015), have reported outbreaks of fire blight disease. While the fire blight disease occurred on apples and pears in 2016 in Ili, Xinjiang, China. These findings support the above proposal that these 15 strains in China and neighboring countries descend from a common ancestor. Additionally, the three Chinese strains with the highest similarity to KAAL06 and KyPL01 were all located in the Ili region (YL16‐01, 1506‐4, 31070‐3). Furthermore, the Khorgos port in the Almaty region (Kazakhstan) is only 15 km away from the Chinese Ili port [14], with international inter‐modal transport capabilities; the source of fire blight in China is probably originated from this area.

### 1.5 *E. amylovora* in China exhibit different pathogenicity

The 15 sequenced strains of *E. amylovora* displayed significant differences in pathogenicity (Figure 2E; Figure S9). While KL17‐17 and KL18‐6 exhibited weak pathogenicity with 0.58% and 4.75% necrosis, respectively, 1506‐4 and 31070‐3 showed very strong pathogenicity with 67.31% and 88.43% necrosis, respectively, on 9 days after inoculation (Figure 2F; Figure S10). However, it is still not clear what causes the decreased pathogenicity in KL17‐17 and KL18‐6 strains. In our future studies, we will employ various approaches, such as transcriptome and metabolome, to reveal the key factors that determine such differences between these strong and weak pathogenic strains.

## AUTHOR CONTRIBUTIONS

**Peijie Gong**: Writing—original draft; methodology; writing—review & editing; visualization; software; project administration; data curation; resources; investigation. **Weibo Sun**: Resources; investigation; data curation. **Jianping Yi**: Resources; formal analysis. **Jian Han**: Resources; methodology; investigation. **Meili Liu**: Data curation; methodology; investigation; resources. **Chenyang Han**: Validation; formal analysis; data curation. **Liang Ming**: Resources; project administration; investigation. **Bangwei Wang**: Methodology; validation; data curation. **Ming Luo**: Resources; data curation. **Yancun Zhao**: Funding acquisition; investigation; methodology; supervision; formal analysis; project administration; resources. **Fengquan Liu**: Project administration; conceptualization; investigation; funding acquisition; methodology; supervision; data curation; resources.

## CONFLICT OF INTEREST STATEMENT

The authors declare no conflicts of interest.

## 1 ETHICS STATEMENT

No animals or humans were involved in this study.

## Supporting information


**Figure S1.** GC Depth distribution map of 15 sequenced *E. amylovora* strains.
**Figure S2.** Distribution map of gene sequence length of 15 *E. amylovora* strains.
**Figure S3.** Comparison the KL20‐28 genome (query genome, y‐axis) with the CFBP1430 genome (reference genome, x‐axis) using the MUMmer tool.
**Figure S4.** The statistical chart of COG annotation for Chinese *E. amylovora* strain KL20‐28 genome.
**Figure S5.** Distribution on of GO function annotation & KEGG function classification of *E. amylovora* strain KL20‐28 genomes.
**Figure S6.** Plasmids identification of 15 sequenced *E. amylovora* isolates in this study.
**Figure S7.** Comparison of the genome synteny among 15 sequenced strains in this study compared with reference strain CFBP1430.
**Figure S8.** Population phylogenetic analysis of *E. amylovora* as circular mode.
**Figure S9.** Colony morphology of 15 *E. amylovora* sequenced strains in this study on nutrient agar (NA).
**Figure S10.** 15 *E. amylovora* stains pathogenicity tests on immature pear fruit on 3, 6 and 9 days after inoculation.
**Figure S11.** Comparative analysis of pathogenic factors in 15 sequenced strains and CFBP1430.

**Table S1.** *E. amylovora* strains used in this study.
**Table S2.** Strains information of 15 whole genome sequenced *E. amylovora* in this study.
**Table S3.** ANIb percentage identity values among 15 sequenced *E. amylovora* and CFBP1430 Strains genomes calculated by JSpecies.
**Table S4.** Statistics of genes from 15 sequenced *E. amylovora* annotated utilizing 4 databases, including Nr, Swiss‐Prot,COG and KEGG.
**Table S5.** COG Functional‐Categories annotation from 15 sequenced *E. amylovora* genomes.
**Table S6.** GO Functional‐Categories annotation from 15 sequenced *E. amylovora* genomes.
**Table S7.** KEGG Functional‐Categories annotation from 15 sequenced *E. amylovora* genomes.
**Table S8.** Plasmids information of 15 *E. amylovora* strains sequenced in this study.
**Table S9**. Plasmid‐1 whole‐sequence alignment descriptions from KL20‐28, KL17‐17, KL18‐6, KL19‐1, ZYGT‐2, 1506‐4, 31070‐3, KyPL01, and KAAL06.
**Table S10.** Plasmid‐2 whole‐sequence alignment descriptions from KL17‐17.
**Table S11.** Genomes alignment 15 sequenced *E. amylovora* strain with the CFBP1430 genome.
**Table S12.** List of SNPs and DIPs present in the genomes of 15 sequenced *E. amylovora* strains, along with their corresponding chromosome positions.
**Table S13.** The download genomes of *E. amylovora* analysed in this study.
**Table S14.** The download genomes of *E. pyrifoliae* analysed in this study.
**Table S15.** Virulence factors of pathogenic bacteria analysis from plasmid pEA29 of 9 *E. amylovora* strains.
**Table S16.** Effects of SNPs called from the alignment of 15 sequenced *E. amylovora* strains genomes compared with CFBP1430.
**Table S17.** Variations in genes associated with bacterial secretion system, flagellar assembly, bacterial chemotaxis, plant‐pathogen interaction and biosynthesis of secondary metabolites of 15 sequenced *E. amylovora* strains genomes compared with CFBP1430a.

## Data Availability

All the data for this project is in the National Genomics Data Center (NGDC) under BioProject accession: PRJCA025867 at https://ngdc.cncb.ac.cn/gsa/. Supplementary materials (methods, figures, tables, graphical abstract, slides, videos, Chinese translated version, and updated materials) may be found in the online DOI or iMeta Science http://www.imeta.science/imetaomics/.
