# Supplementary material for: Comparative genomic analysis of Chinese Erwinia amylovora strains reveals genetic variations, plasmid diversity, and potential common ancestor
Source: IMetaOmics. 2024 Nov 29;2(1):e44. doi: 10.1002/imo2.44 (PMC12806430; doi:10.1002/imo2.44)
Supplement: Supplementary file 1 — Figure S1. GC Depth distribution map of 15 sequenced E. amylovora strains. Figure S2. Distribution map of gene sequence length of 15 E. amylovora strains. Figure S3. Comparison the KL20‐28 genome (query genome, y‐axis) with the CFBP1430 genome (reference genome, x‐axis) using the MUMmer tool. Figure S4. The statistical chart of COG annotation for Chinese E. amylovora strain KL20‐28 genome. Figure S5. Distribution on of GO function annotation & KEGG function classification of E. amylovora strain KL20‐28 genomes. Figure S6. Plasmids identification of 15 sequenced E. amylovora isolates in this study. Figure S7. Comparison of the genome synteny among 15 sequenced strains in this study compared with reference strain CFBP1430. Figure S8. Population phylogenetic analysis of E. amylovora as circular mode. Figure S9. Colony morphology of 15 E. amylovora sequenced strains in this study on nutrient agar (NA). Figure S10. 15 E. amylovora stains pathogenicity tests on immature pear fruit on 3, 6 and 9 days after inoculation. Figure S11. Comparative analysis of pathogenic factors in 15 sequenced strains and CFBP1430. [file IMO2-2-e44-s002.docx]

Supporting information to

Comparative genomic analysis of Chinese Erwinia amylovora strains reveals genetic variations, plasmid diversity, and potential common ancestor

**Running title**: Comparative genomic analysis of *Erwinia amylovora* in China

Peijie Gong^1^, Weibo Sun^1^, Jianping Yi^3^, Jian Han^4^, Meili Liu^1^, Chenyang Han^5^, Liang Ming^1^, Bangwei Wang^1,6^, Ming Luo^4^, Yancun Zhao^1,4*^, Fengquan Liu^1,2*^

^1^Institute of Plant Protection, Jiangsu Academy of Agricultural Sciences, Jiangsu Key Laboratory for Food Quality and Safety, State Key Laboratory Cultivation Base of Ministry of Science and Technology, Nanjing 210014, China

^2^Department of Plant Pathology/Key Laboratory of Agricultural Microbiology, College of Agriculture, Guizhou University, Guiyang 550025, China

^3^Technical Center for Animal, Plant and Food Inspection and Quarantine of Shanghai Customs, Shanghai 200135, China

^4^College of Agronomy, Xinjiang Agriculture University, Wulumuqi 830052, China

^5^Center of Pear Engineering Technology Research, State Key Laboratory of Crop Genetics and Germplasm Enhancement, College of Horticulture, Nanjing Agricultural University, Nanjing 210095, China

^6^College of Plant Protection, Nanjing Agricultural University, Nanjing 210095, China

^*^Correspondence: [zhaoyc27@126.com](mailto:zhaoyc27@126.com) (Yancun Zhao); [fqliu20011@sina.com](mailto:fqliu20011@sina.com) (Fengquan Liu)

**LIST OF SUPPLEMENTAL INFORMATION**

**Supplemental Methods**

**Supplemental Figures**

**Figure S1.** GC Depth distribution map of 15 sequenced *E.amylovora* strains.

**Figure S2.** Distribution map of gene sequence length of 15 *E.amylovora* strains.

**Figure S3.** Comparison the KL20-28 genome (query genome, y-axis) with the CFBP 1430 genome (reference genome, x-axis) using the MUMmer tool.

**Figure S4.** The statistical chart of COG annotation for Chinese *E. amylovora* strain KL20-28 genome.

**Figure S5.** Distribution on of GO function annotation & KEGG function classification of *E. amylovora* strain KL20-28 genomes.

**Figure S6.** Plasmids identification of 15 sequenced *E.amylovora* isolates in this study.

**Figure S7.** Comparison of the genome synteny among 15 sequenced strains in this study compared with reference strain CFBP 1430.

**Figure S8.** Population phylogenetic analysis of *E. amylovora* as circular mode.

**Figure S9.** Colony morphology of 15 *Erwinia amylovora* sequenced strains in this study on nutrient agar (NA).

**Figure S10.** 15 *Erwinia amylovora* stains pathogenicity tests on immature pear fruit on 3, 6 and 9 days after inoculation.

**Figure S11.** Comparative analysis of pathogenic factors in 15 sequenced strains and CFBP1430.

**Material and methods**

**Bacterial strains and plant materials**

The *E. amylovora* strains involved in this study are shown in Table S1. NB medium (peptone 5 g/L, sucrose 10 g/L, yeast extract 1 g/L, beef extract 3 g/L, pH adjusted to 6.8-7.2) was utilized for routine growth of each strains. The experimental pear varieties in this study is *Pyrus* cv. ‘Zaosu’.

**Whole genome sequencing and assembly**

*E. amylovora* bacterial genome sequencing using a combination of third-generation PacBio and second-generation Illumina sequencing platform by Genedenovo Biotechnology Co., Ltd (Guangzhou, China). Relying on the characteristics of PacBio long reading to ensure more complete genome assembly. Illumina is used to correct the data to ensure more accurate and reliable assembly results.

To ensure the quality of sequencing, We utilized the Bacterial DNA Extraction Kit (TIANGEN, China) to extract high-quality DNA from the bacterial strains in this study. And DNA quality of each sample was tested by using Qubit as a standard, and using Nanodrop as an auxiliary test (OD260/280 between 1.8-2.0 required). The instruments used for PacBio sequencing are primarily RSII and Sequel (Pacific Biosciences, USA). The genomic libraries were constructed using G-tubes method to process genomic DNA into 8-10k fragments. Further fragment selection of each library was performed using the nucleic acid fragment recovery system BluePippin (BP) . Once the library is built, Qubit is used for quality inspection and the Agilent 2100 was used to evaluate the size of the insert. Sequencing and assembling processes were then performed using the PacBio platform. The assembly results are then corrected using second-generation Illumina data. Genome component analysis, functional annotation, comparative genomic analysis, etc. based on corrected assembly results. Afterwards, DIAMOND and COG (Cluster of Orthologous Groups of proteins) were used for database comparison to obtain gene corresponding annotation results. Proteins were functionally classified based on the annotation results. The COG database is divided into 26 categories by function（http://www.ncbi.nlm.nih.gov/COG/）.

**Comparative genomics analysis**

In order to compare the evolutionary genomics difference between Chinese strains (represented by KL 20-28) and international model strains, CFBP 1430 (isolated from France) was selected as the reference genome. The target genome and reference genome were compared using MUMmer software by collinearity analysis method to determine the large-scale collinear relationship between genomes [[1](#_ENREF_1)]. Then software SyRI was used to compare the regions[[2](#_ENREF_2)], confirm the local position arrangement relationship, and find the regions of translocation/Trans, Inversion/Inv and Trans+Inv.

**ANI (Average Nucleotide Identity) analysis**

Pyani method was used to calculate the average nucleotide homology ANI value of the bigenomic alignment region of the target genome and the reference related genome [[3](#_ENREF_3)]. Further, the BLASTN-based average nucleotide homology value was calculated using the JSpecies model to assess similarities between genome sequences [[4](#_ENREF_4)]. Heat maps are used to cluster and visualize similar value matrices. The ANI value distinguishes different species with 95% as the classification threshold.

**Detection and analysis of genomic structural variations in *E.amylovora* strains**

For the strains ZY20-3-2, KL17-17, KL20-28, and other strains, the genomic structural variations, including SNPs (single-nucleotide polymorphisms) and DIPs (short deletions, insertions, and other polymorphisms) were detected. The MUMmer alignment software was used to detect and compare SNPs and DIPs in the assembled genomes of the individual strains. The positions and mutation results between genes based on SNPs or DIPs were statistically analyzed. Furthermore, the genomic sequences of the European isolate CFBP 1430 were compared with the genomes of the Chinese indigenous strains mentioned above. The SNPs, DIPs, and other polymorphisms were explored using whole-genome alignment and the TRAMS software [[5](#_ENREF_5)]. Subsequently, the software was used to annotate the variant positions and predict the impact of sequence changes on the functionality of the associated genes.

**Plasmid identification and diversity assay**

Plasmid sequencing was performed simultaneously with genome sequencing. After obtaining the presence and sequence information of plasmids in each strain, similarity to plasmids in the GenBank database was compared. The specific method involved assembling the sequences of DNA contigs that could not be concatenated with the entire genome, analyzing the number and coverage of closed circular assemblies, and conducting comparative analysis. Sequences with coverage greater than one standard deviation from the median were compared for similarity to known plasmids, including the *Erwinia* plasmids and those in the GenBank database [[6](#_ENREF_6)]. If a novel plasmid was found through the alignment, it was named according to the plasmid identified result [[7](#_ENREF_7)].

**Genomic diversity and phylogenetic tree construction**

A multiple sequence alignment was performed between the whole genome sequences of 97 American, 9 Asian, 22 European, 2 African, 2 Oceanian *E. amylovora* strains and 10 *E. pyrifoliae* (fire blight disease in Asia), along with 13 Chinese , 1 Kazakhstan and 1 Kyrgyzstan isolates[[7-18](#_ENREF_7)] (Table S13-S14) . The genomes of all strains, except for the 15 strain isolated and sequenced in this study, were downloaded from NCBI (https://www.ncbi.nlm.nih.gov/datasets/genome). Phylogenetic analysis was performed to generate an ML phylogenetic tree with 1,000 bootstraps using 2120 single copy orthologous genes [[19](#_ENREF_19)], conserved among 130 species using the Orthofinder2 software and ITOL (https://itol.embl.de/).

***E. amylovora* pathogenicity test on *Pyrus bretschneideri* immature fruits**

Virulence assessments on immature 'Zaosu' pear fruits were conducted as previously detailed[[20](#_ENREF_20)]. In summary, the bacteria were cultivated in NB medium overnight, subsequently collected via centrifugal, and rinsed with ddH_2_O 3 times. Then the bacteria pellet was re-suspended in ddH_2_O (OD600= 0.1 and then diluted 100-fold). The immature pears were subjected to surface sterilization and punctured with a sterile needle. The punctured fruits were then inoculated with 2 µl of the bacterial suspension and maintained at a relative humidity of 100% at a temperature of 28℃. The symptoms were documented at the five-day post-inoculation. For each strain, the process was replicated with three fruits. The virulence was quantitatively assessed by calculating the percentage of necrosis, which is the ratio of the necrotic and water-soaked tissue surface area to the total pear surface area.

**Data Availability**

The relevant data of sequenced 15 *E. amylovora* isolates in this study (KL20-28, KL17-17, ZY20-3-2, AKS17-22, KL18-6, KL19-1, KL21-15, KS22-2, YL16-01, YL16-15, ZYGT-2, 1506-4, 31070-3, KAAL06 and KyPL01) have been deposited in the National Genomics Data Center (NGDC)[[21](#_ENREF_21)], Beijing Institute of Genomics, Chinese Academy of Sciences/China National Center for Bioinformation, under the BioProject accession number [PRJCA025867](https://ngdc.cncb.ac.cn/gsub/submit/bioproject/PRJCA025867) that is publicly accessible at https://ngdc.cncb. ac.cn/gwh. The raw Illumina NovaSeq 6000 genome sequencing data reported in this paper have been deposited in the Genome Sequence Archive[[22](#_ENREF_22)] in NGDC, China National Center for Bioinformation/Beijing Institute of Genomics, Chinese Academy of Sciences (GSA: CRA016397) that are publicly accessible at <https://ngdc.cncb.ac.cn/gsa>, under the accession number CRR1144183^29^, CRR1144184^30^, CRR1144185^31^, CRR1144186^32^, CRR1144187^33^, CRR1144188^34^, CRR1144189^35^, CRR1144190^36^, CRR1144191^37^, CRR1144192^38^, CRR1144193^39^, CRR1144194^40^, CRR1144195^41^, CRR1144196^42^, CRR1144197^43^.

**Main references**

1. Kurtz, Stefan, Adam Phillippy, Arthur L. Delcher, Michael Smoot, Martin Shumway, Corina Antonescu, Steven L. Salzberg. 2004. “Versatile and open software for comparing large genomes.” *Genome Biology* 5: R12. https://doi.org/10.1186/gb-2004-5-2-r12

2. Goel, Manish, Hequan Sun, Wen-Biao Jiao, Korbinian Schneeberger. 2019. “SyRI: finding genomic rearrangements and local sequence differences from whole-genome assemblies.” *Genome Biology* 20: 277. https://doi.org/10.1186/s13059-019-1911-0

3. Pritchard, Leighton, Rachel Glover, Sonia Humphris, J. Elphinstone, Ian Toth. 2015. “Genomics and Taxonomy in Diagnostics for Food Security: soft-rotting enterobacterial plant pathogens.” *Anal. Methods* 8: https://doi.org/10.1039/C5AY02550H

4. Richter, M., R. Rosselló-Móra. 2009. “Shifting the genomic gold standard for the prokaryotic species definition.” *Proc Natl Acad Sci U S A* 106: 19126-19131. https://doi.org/10.1073/pnas.0906412106

5. Reumerman, Richard A., Nicholas P. Tucker, Paul R. Herron, Paul A. Hoskisson, Vartul Sangal. 2013. “Tool for rapid annotation of microbial SNPs (TRAMS): a simple program for rapid annotation of genomic variation in prokaryotes.” *Antonie Van Leeuwenhoek* 104: 431-434. https://doi.org/10.1007/s10482-013-9953-x

6. Clark, Karen, Ilene Karsch-Mizrachi, David J. Lipman, James Ostell, Eric W. Sayers. 2016. “GenBank.” *Nucleic Acids Res* 44: D67-D72. https://doi.org/10.1093/nar/gkv1276

7. Parcey, M., S. Gayder, V. Morley-Senkler, G. Bakkeren, J. R. Úrbez-Torres, S. Ali, A. J. Castle, A. M. Svircev. 2020. “Comparative genomic analysis of Erwinia amylovora reveals novel insights in phylogenetic arrangement, plasmid diversity, and streptomycin resistance.” *Genomics* 112: 3762-3772. https://doi.org/10.1016/j.ygeno.2020.04.001

8. Boulé, J., P. L. Sholberg, S. M. Lehman, D. T. O'Gorman, A. M. Svircev. 2011. “Isolation and characterization of eight bacteriophages infecting Erwinia amylovora and their potential as biological control agents in British Columbia, Canada.” *Canadian Journal of Plant Pathology* 33: 308-317. https://doi.org/10.1080/07060661.2011.588250

9. Mann, Rachel A., Theo H. M. Smits, Andreas Bühlmann, Jochen Blom, Alexander Goesmann, Jürg E. Frey, Kim M. Plummer, et al. 2013. “Comparative Genomics of 12 Strains of Erwinia amylovora Identifies a Pan-Genome with a Large Conserved Core.” *PLoS One* 8: e55644. https://doi.org/10.1371/journal.pone.0055644

10. Smits, T. H., F. Rezzonico, T. Kamber, J. Blom, A. Goesmann, J. E. Frey, B. Duffy. 2010. “Complete genome sequence of the fire blight pathogen Erwinia amylovora CFBP 1430 and comparison to other Erwinia spp.” *Mol Plant Microbe Interact* 23: 384-393. https://doi.org/10.1094/mpmi-23-4-0384

11. Smits, T. H., V. M. Guerrero-Prieto, G. Hernández-Escarcega, J. Blom, A. Goesmann, F. Rezzonico, B. Duffy, V. O. Stockwell. 2014. “Whole-Genome Sequencing of Erwinia amylovora Strains from Mexico Detects Single Nucleotide Polymorphisms in rpsL Conferring Streptomycin Resistance and in the avrRpt2 Effector Altering Host Interactions.” *Genome Announc* 2: https://doi.org/10.1128/genomeA.01229-13

12. Zeng, Q., Z. Cui, J. Wang, K. L. Childs, G. W. Sundin, D. R. Cooley, C. H. Yang, et al. 2018. “Comparative genomics of Spiraeoideae-infecting Erwinia amylovora strains provides novel insight to genetic diversity and identifies the genetic basis of a low-virulence strain.” *Mol Plant Pathol* 19: 1652-1666. https://doi.org/10.1111/mpp.12647

13. Sebaihia, M., A. M. Bocsanczy, B. S. Biehl, M. A. Quail, N. T. Perna, J. D. Glasner, G. A. DeClerck, et al. 2010. “Complete genome sequence of the plant pathogen Erwinia amylovora strain ATCC 49946.” *J Bacteriol* 192: 2020-2021. https://doi.org/10.1128/jb.00022-10

14. Evans, C. K.2008. SURVEY RESULTS OF ERWINIA AMYLOVORA IN UTAH FOR RESISTANCE TO STREPTOMYCIN AND INVESTIGATIONS COMPARING KASUGAMYCIN (KASUMIN) TO STREPTOMYCIN AND OXYTETRACYCLINE FOR CONTROL OF FIRE BLIGHT:433-437.

15. Song, J. Y., Y. H. Yun, G. D. Kim, S. H. Kim, S. J. Lee, J. F. Kim. 2021. “Genome Analysis of Erwinia amylovora Strains Responsible for a Fire Blight Outbreak in Korea.” *Plant Dis* 105: 1143-1152. https://doi.org/10.1094/pdis-06-20-1329-re

16. Zrelovs, N., A. Dislers, A. Kazaks. 2020. “Novel Erwinia persicina Infecting Phage Midgardsormr38 Within the Context of Temperate Erwinia Phages.” *Front Microbiol* 11: 1245. https://doi.org/10.3389/fmicb.2020.01245

17. Zhao, Youfu, Mingsheng Qi. 2011. 'Comparative Genomics of Erwinia amylovora and Related Erwinia Species—What do We Learn?', *Genes (Basel)*, pp. 627-639.

18. Smits, Theo H. M., Sebastian Jaenicke, Fabio Rezzonico, Tim Kamber, Alexander Goesmann, Jürg E. Frey, Brion Duffy. 2010. “Complete genome sequence of the fire blight pathogen Erwinia pyrifoliae DSM 12163T and comparative genomic insights into plant pathogenicity.” *BMC Genomics* 11: 2. https://doi.org/10.1186/1471-2164-11-2

19. Nguyen, Lam-Tung, Heiko A. Schmidt, Arndt von Haeseler, Bui Quang Minh. 2015. “IQ-TREE: A Fast and Effective Stochastic Algorithm for Estimating Maximum-Likelihood Phylogenies.” *Molecular Biology and Evolution* 32: 268-274. https://doi.org/10.1093/molbev/msu300

20. McNally, R. R., I. K. Toth, P. J. Cock, L. Pritchard, P. E. Hedley, J. A. Morris, Y. Zhao, G. W. Sundin. 2012. “Genetic characterization of the HrpL regulon of the fire blight pathogen Erwinia amylovora reveals novel virulence factors.” *Mol Plant Pathol* 13: 160-173. https://doi.org/10.1111/j.1364-3703.2011.00738.x

21. Partners, CNCB-NGDC Members and. 2022. “Database Resources of the National Genomics Data Center, China National Center for Bioinformation in 2022.” *Nucleic Acids Res* 50: D27-D38. https://doi.org/10.1093/nar/gkab951

22. Chen, Tingting, Xu Chen, Sisi Zhang, Junwei Zhu, Bixia Tang, Anke Wang, Lili Dong, et al. 2021. “The Genome Sequence Archive Family: Toward Explosive Data Growth and Diverse Data Types.” *Genomics, Proteomics & Bioinformatics* 19: 578-583. https://doi.org/https://doi.org/10.1016/j.gpb.2021.08.001

**Supplemental Figures**

**
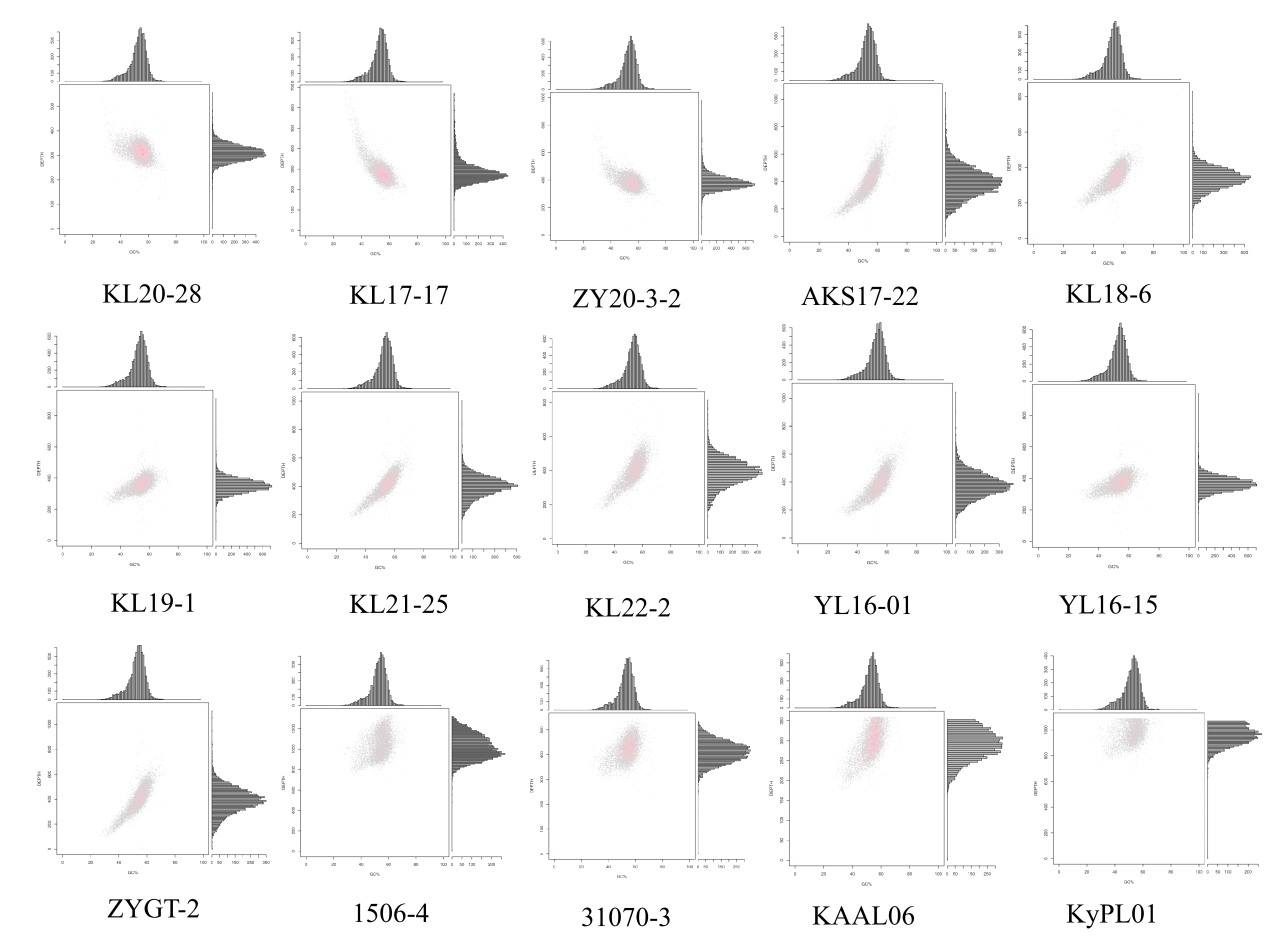
**

**Figure S1.** GC Depth distribution map of 15 sequenced *E.amylovora* strains. The horizontal axis represents GC content, the vertical axis represents sequencing depth, the distribution of sequencing depth is on the right, and the distribution of GC content is on the top. Due to the varying GC content of each species, the GC content of the same species will exhibit a concentrated distribution. If the majority of points are concentrated within a relatively narrow range, it indicates that there is no species pollution.

**
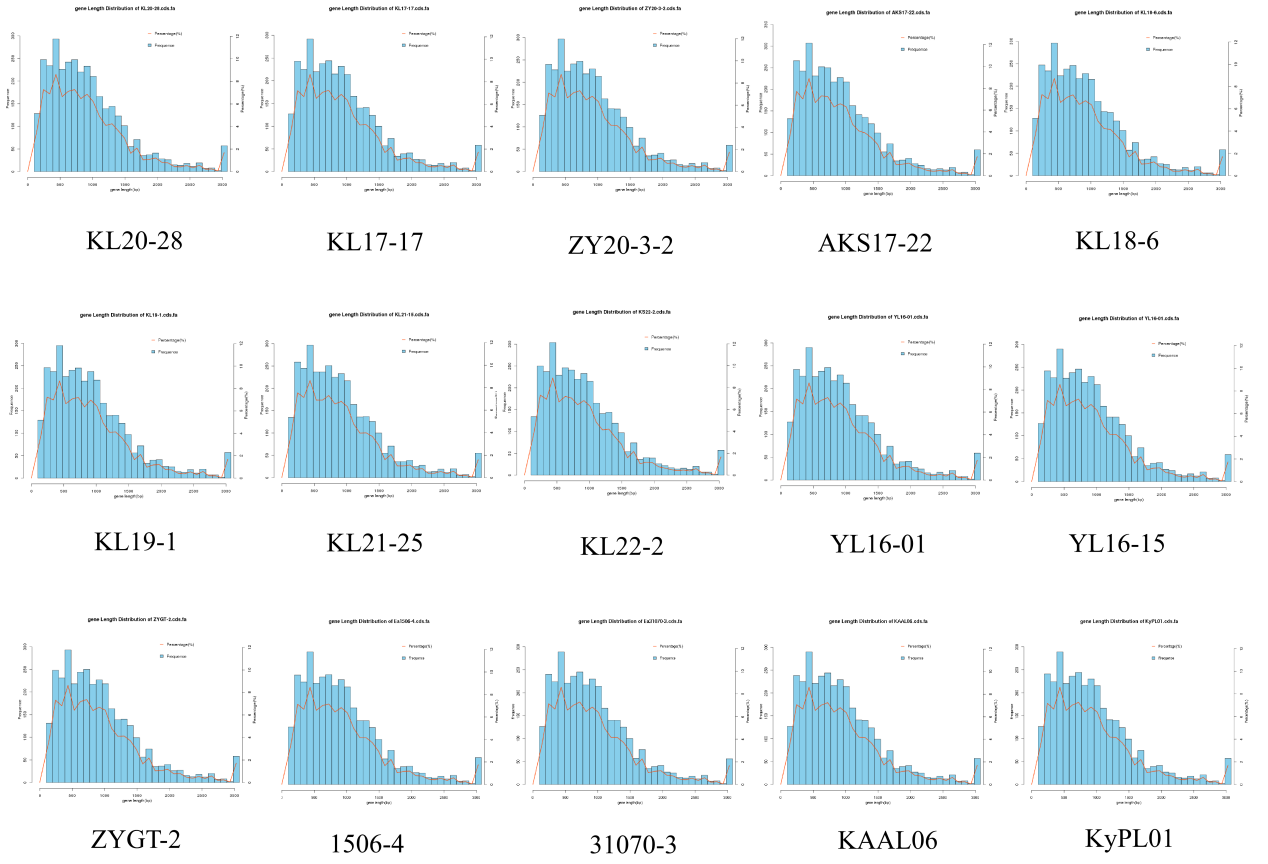
**

**Figure S2.** Distribution map of gene sequence length of 15 *E.amylovora* strains. The horizontal axis represents the predicted gene length; The vertical axis (left) corresponds to a bar chart, representing the number of genes of that length; The vertical axis (right) corresponds to a line graph, representing the percentage of genes of this length in the total number of genes.

**
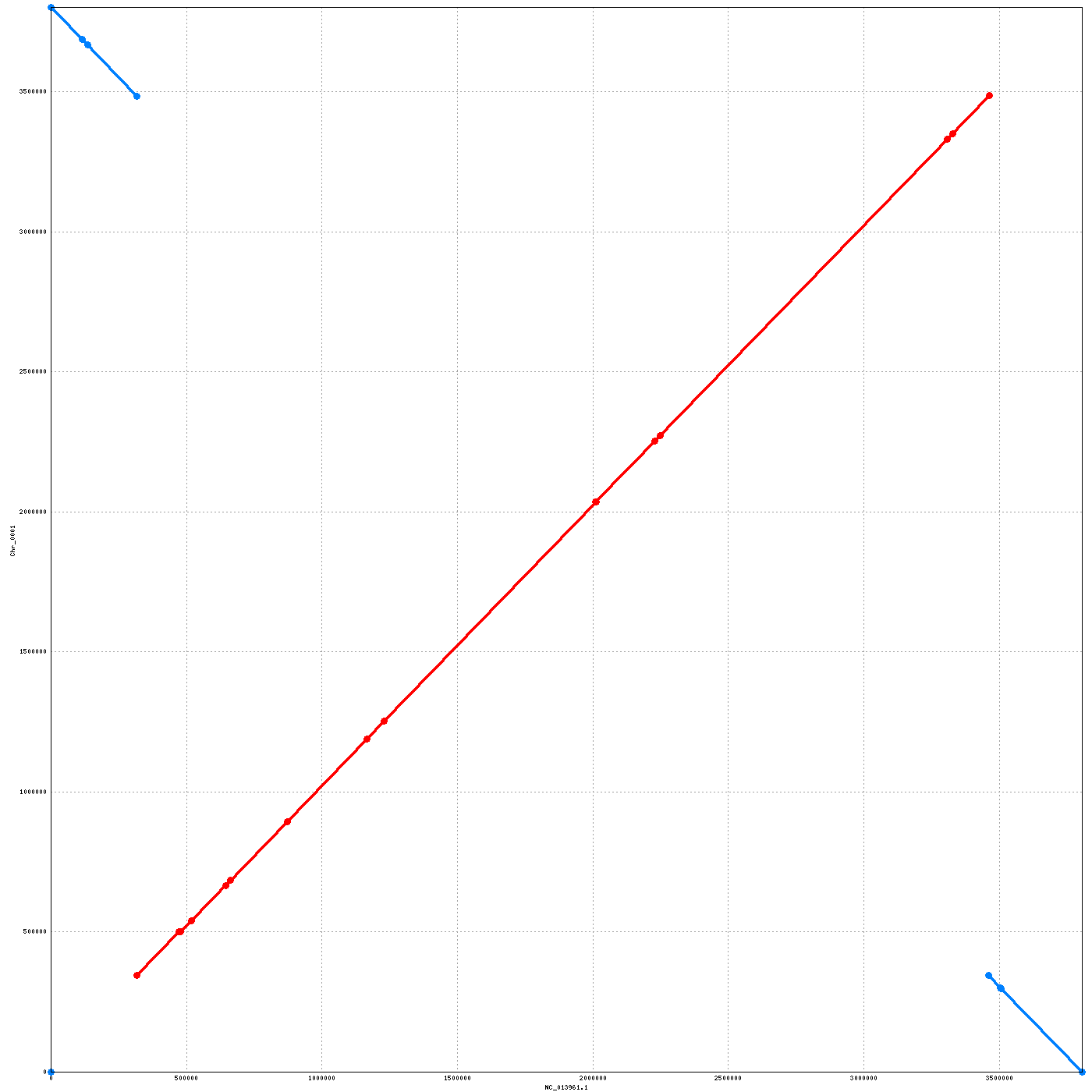
**

**Figure S3.** Comparison the KL20-28 genome (query genome, y-axis) with the CFBP 1430 genome (reference genome, x-axis) using the MUMmer tool. Red indicates forward matches, blue indicates reverse matches.


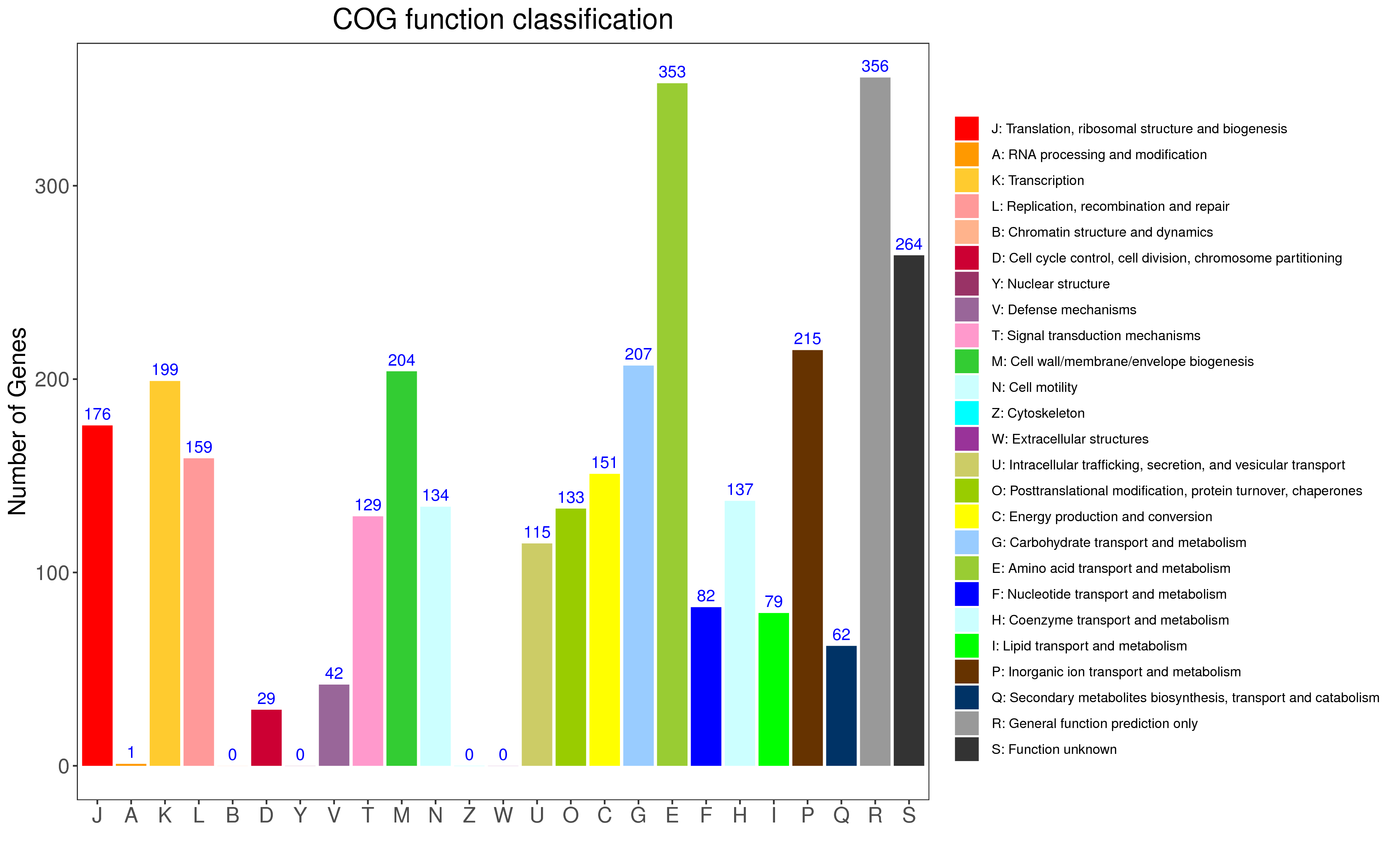


**Figure S4.** The statistical chart of COG annotation for Chinese *E. amylovora* strain KL20-28 genome. The horizontal axis represents the functional categories, which are displayed in various colors; The vertical axis indicates the number of predicted genes contained within each functional classification.


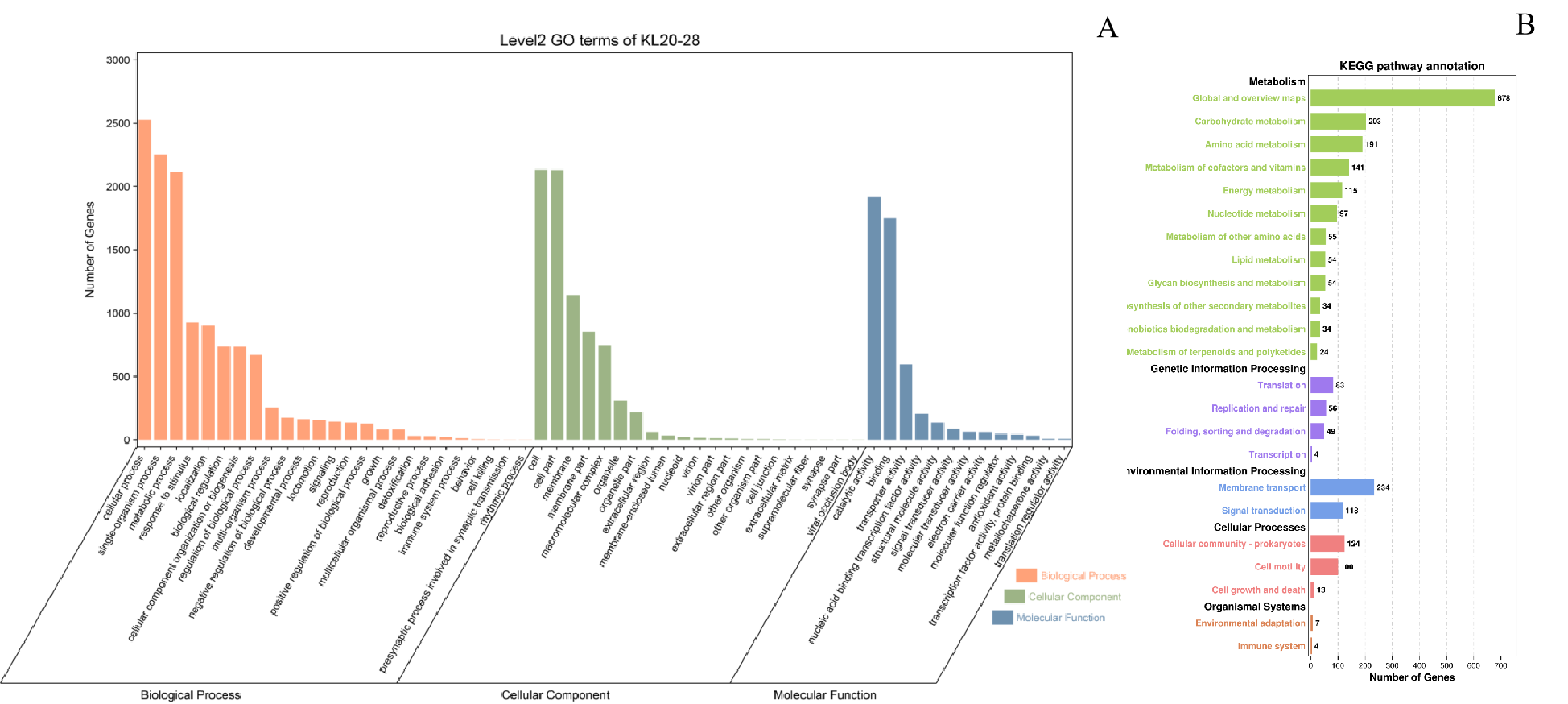


**Figure S5.** Distribution on of GO function annotation & KEGG function classification of *E.amylovora* strain KL20-28 genomes.

1. The vertical axis represents functional classification, cellular component (the parts of a cell or its extracellular environment), molecular function (the elemental activities of a gene product at the molecular level, such as binding or catalysis) and biological process (operations or sets of molecular events with a defined beginning and end, pertinent to the functioning of integrated living units: cells, tissues, organs, and organisms) displayed in red, green and blue colors respectively, while the horizontal axis represents the number of predicted genes included in each functional classification. B. The KEGG annotation information was used to generate a statistical analysis diagram of gene metabolic pathway functional classification. The vertical axis represents functional classification, with Metabolism, Genetic Information Processing, Environmental Information Processing, Cellular Processes, and Organism Systems shown in green, purple, blue, red, and orange, respectively. The horizontal axis represents the number of predicted genes included in each functional classification.


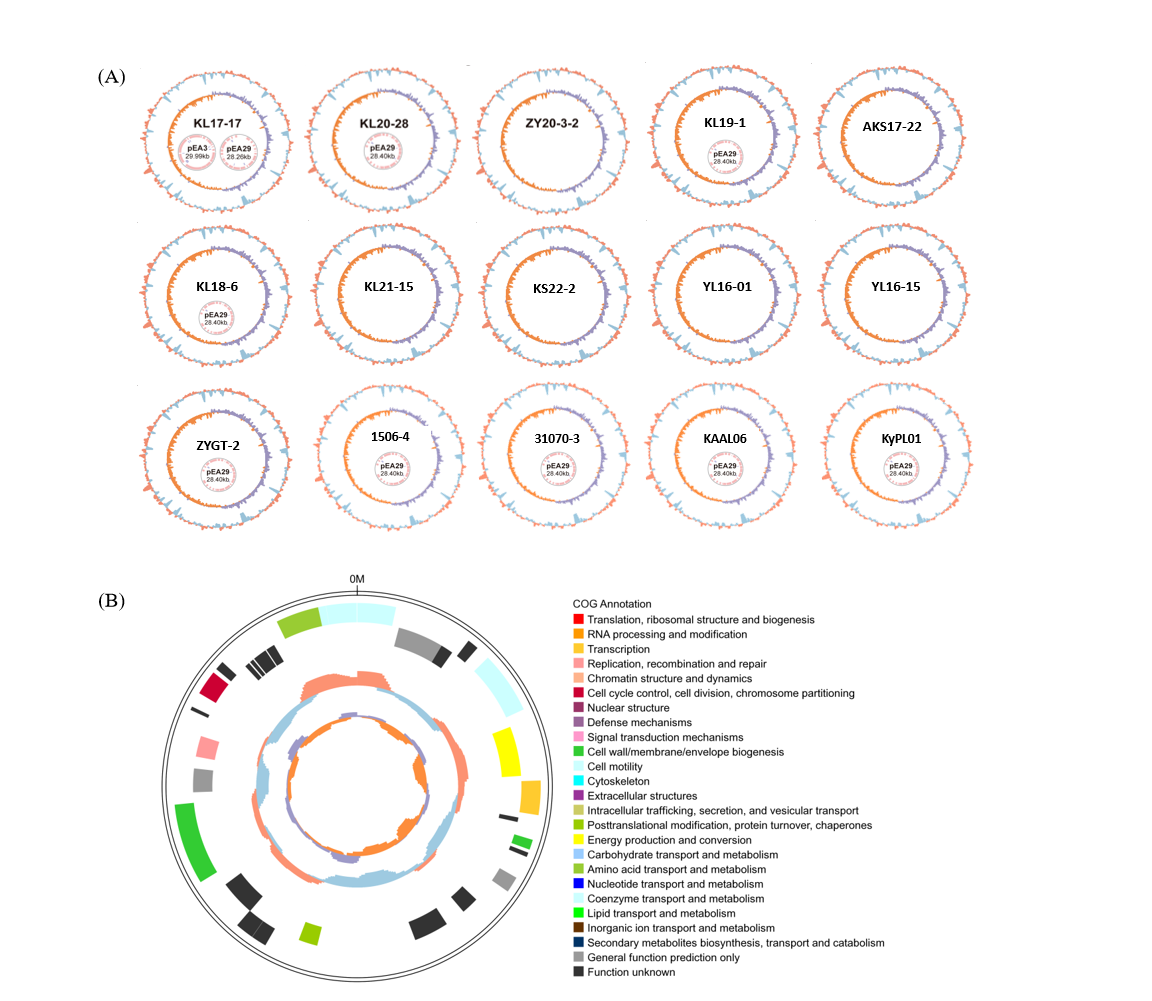


**Figure S6.** Plasmids identification of 15 sequenced *E.amylovora* isolates in this study. (A) Distribution of plasmids in different strains. (B) *pEA29* plasmid circle diagram, a comprehensive display of genomic features, such as the distribution of genes on the ortho and antisense strands, the COG functional classification of genes, and the GC content (red indicates above the mean, blue indicates below the mean), and GC skew (used to measure the relative content of G and C in a circular chromosome, marking the start and endpoints; GC skew = (G-C)/(G+C); purple indicates above 0, orange indicates below 0.


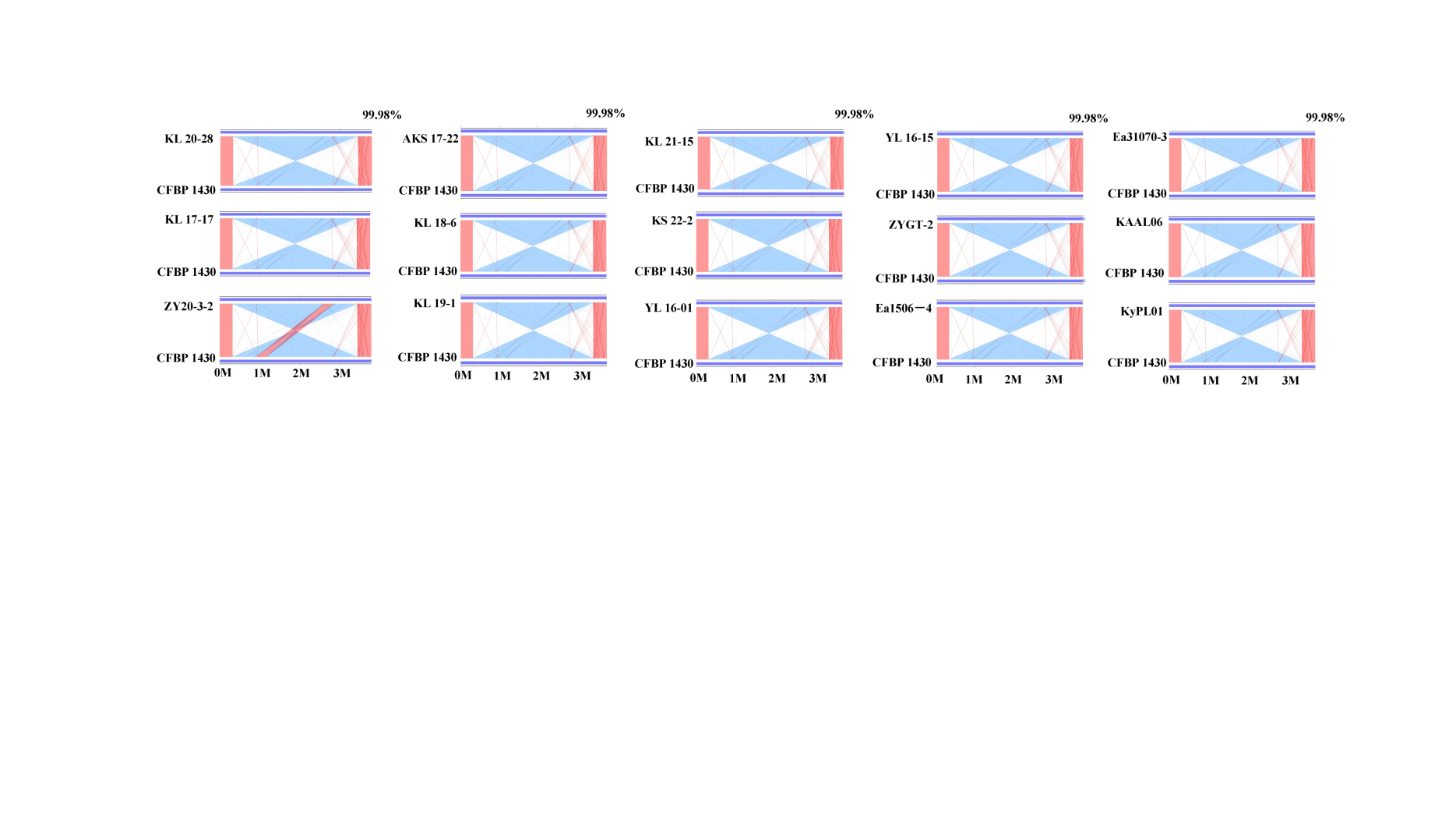


**Figure S7.** Comparison of the genome synteny among 15 sequenced strains in this study compared with reference strain CFBP 1430. Linear and inverted relationships between the genomic sequences are indicated by red and blue, respectively.


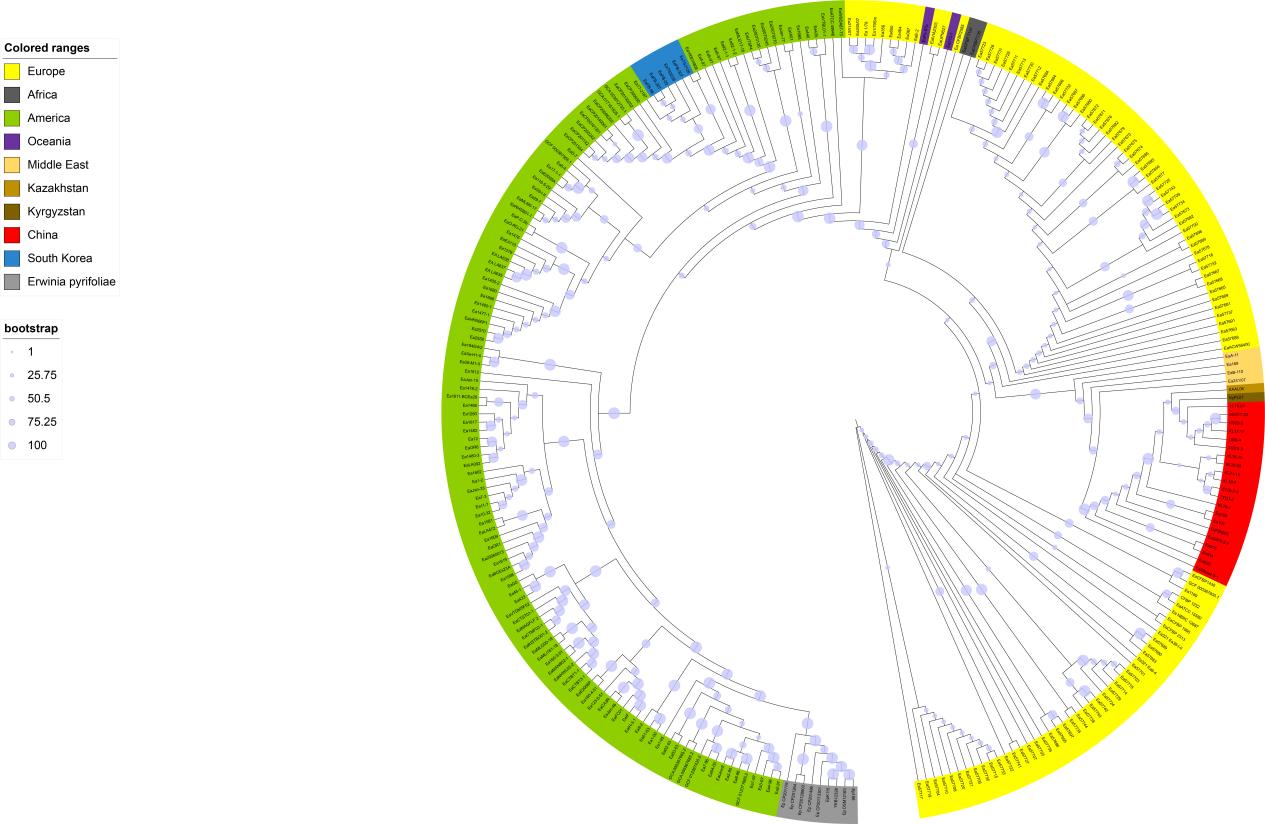


**Figure S8.** Population phylogenetic analysis of *E. amylovora* as circular mode. Phylogenetic circular tree revealed the relationship of 15 *E. amylovora* strains in this study among with other 255 *E. amylovora* strains and 9 *E. pyrifoliae* from various parts of the globe, using OrthoFinder2 through 2120 single copy orthologous genes of each stain using ITOL software.


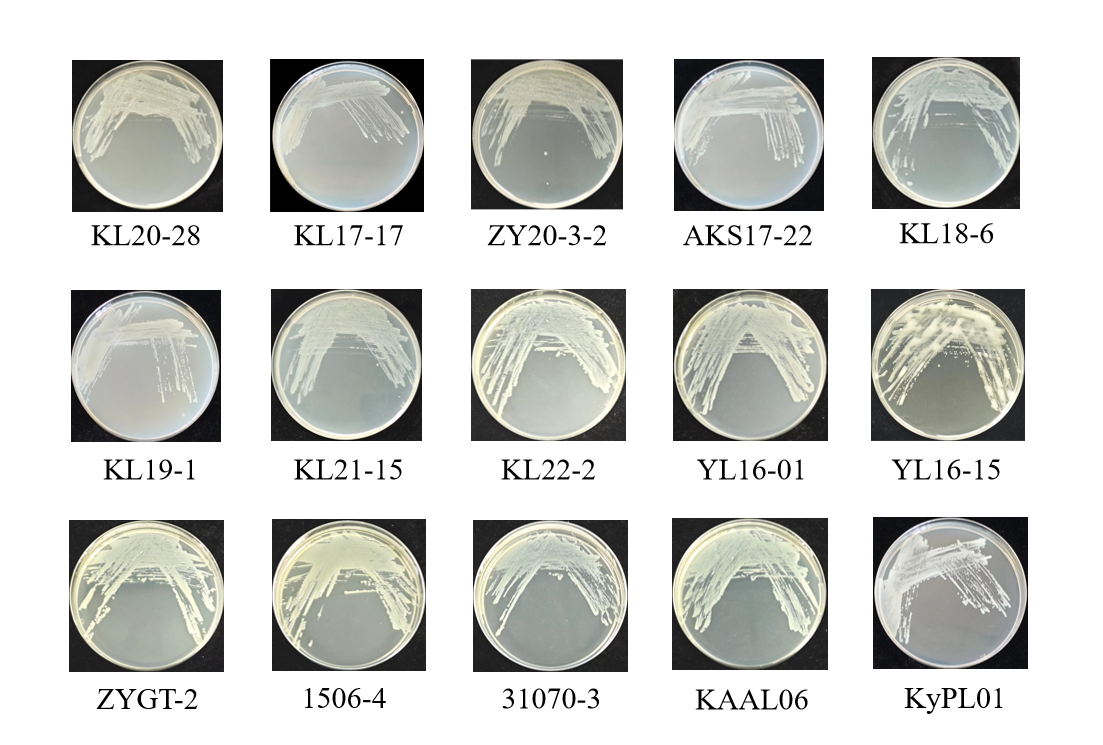


**Figure S9.** Colony morphology of 15 *Erwinia amylovora* sequenced strains in this study on nutrient agar (NA). The strains’ names of morphological colony are KL20-28、KL17-17、ZY20-3-2、AKS17-22、KL18-6、KL19-1、KL21-15、KS22-2、YL16-01、YL16-15、ZYGT-2、1506-4、31070-3、KAAL06、KyPL01, respectively.


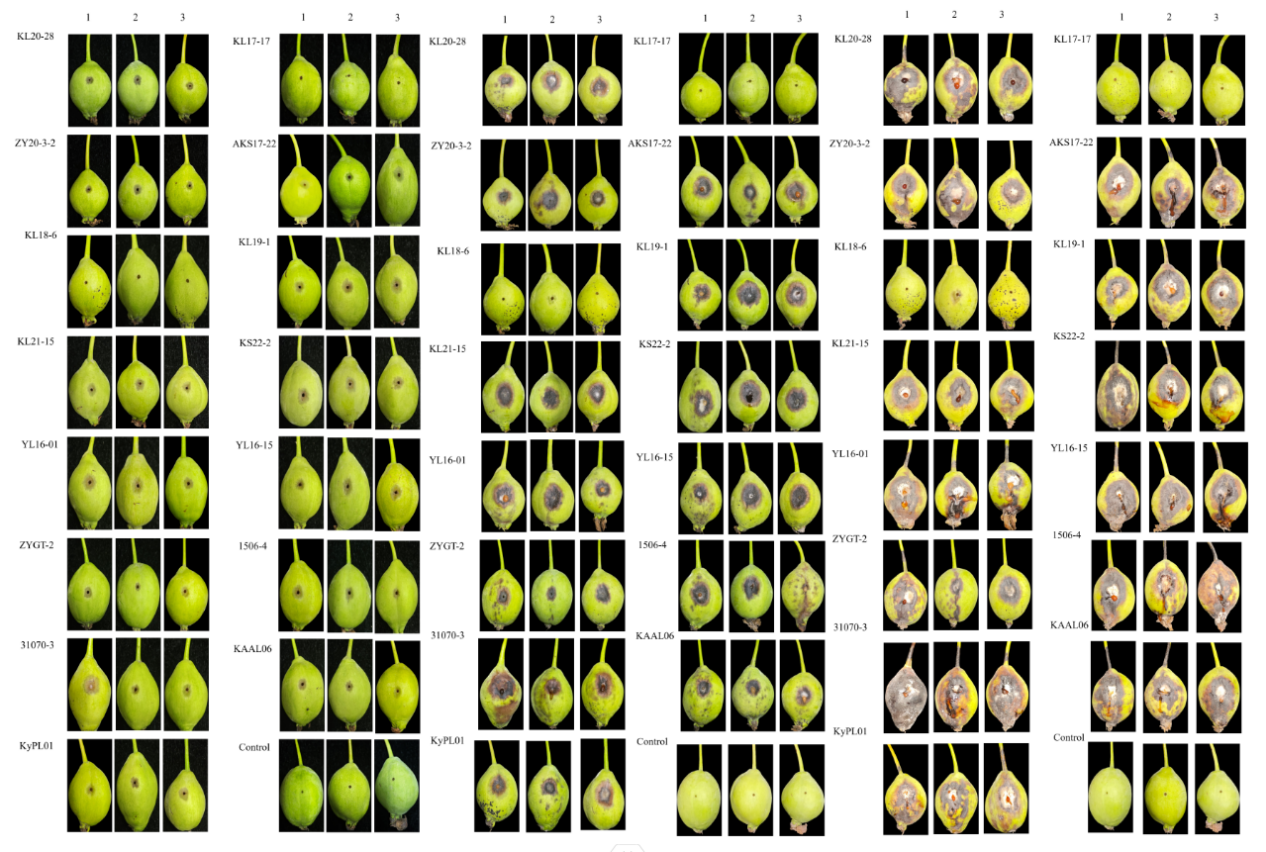


**Figure S10. 15 *Erwinia amylovora* stains pathogenicity tests on immature pear fruit on 3, 6 and 9 days after inoculation.** Symptoms caused by the each *E.amylovora* stain KL20-28, KL17-17, ZY20-3-2，AKS17-22, KL18-6, KL19-1, KL21-15, KS22-2, YL16-01, YL16-15, ZYGT-2, 31070-3, 1506-4, KAAL06 and KyPL01.


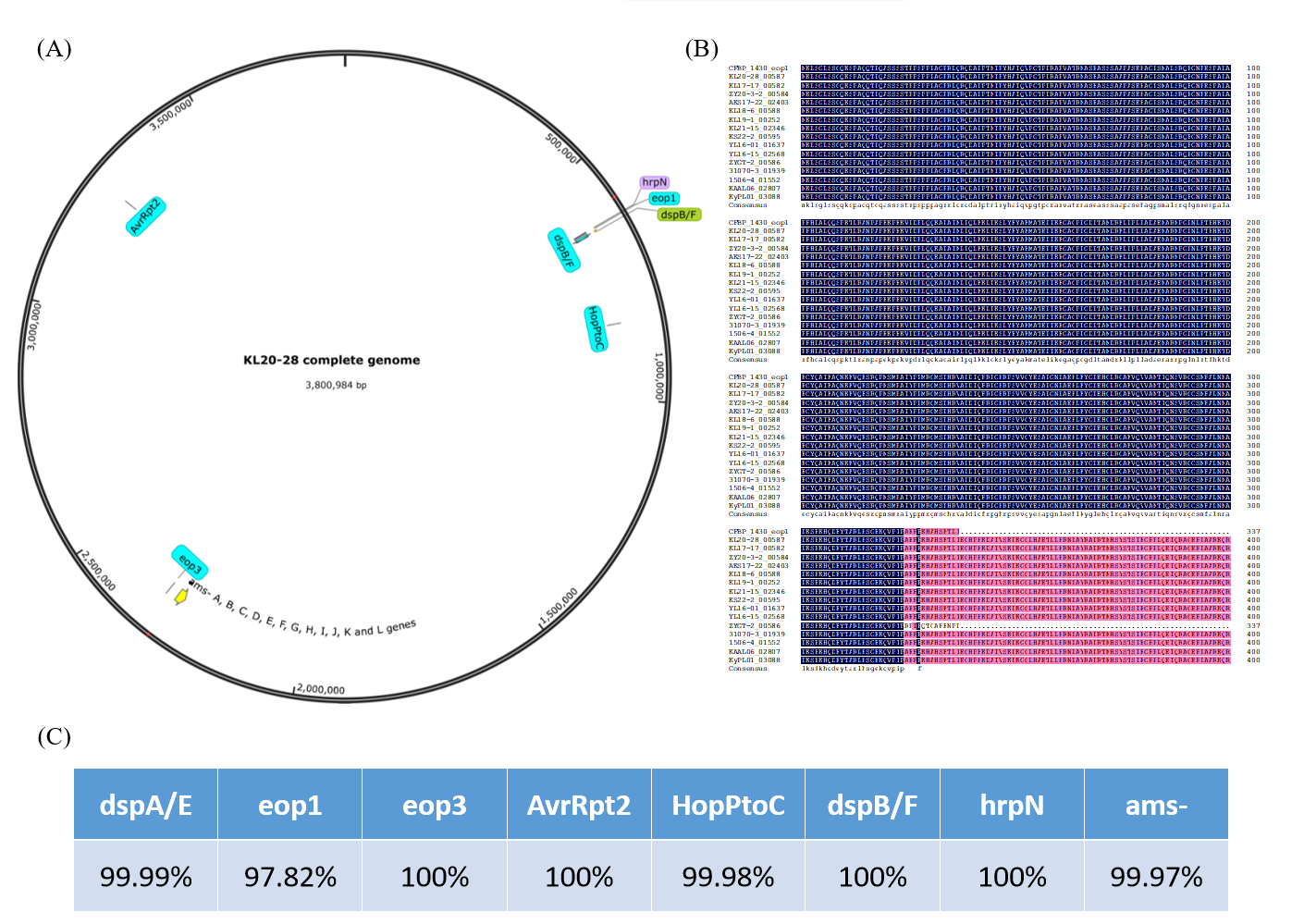


**Figure S11. Comparative analysis of pathogenic factors in 15 sequenced strains and CFBP1430.**

(A) Annotated map of key factors related to pathogenicity on KL20-28 whole genome, including effectors dspA/E, eop1, eop3, AvrRpt2, and HopPtoC; Harpin protein hrpN; Chaperone protein dspB/F; amylovoran synthesis cluster ams- A, B, C, D, E, F, G, H, I, J, K and L genes. (B) Alignment of eop1 amino acid sequences in 15 strains and CFBP 1430. (C) Identity valute among pathogenicity related protein from 15 sequenced strains and CFBP 1430.
